# Novel Diphenylamine Analogs Induce Mesenchymal to Epithelial Transition in Triple Negative Breast Cancer

**DOI:** 10.3389/fonc.2019.00672

**Published:** 2019-07-30

**Authors:** Akshita B. Bhatt, Mohit Gupta, Van T. Hoang, Suravi Chakrabarty, Thomas D. Wright, Steven Elliot, Ishveen K. Chopra, Darlene Monlish, Katie Anna, Matthew E. Burow, Jane E. Cavanaugh, Patrick T. Flaherty

**Affiliations:** ^1^Division of Pharmacology, School of Pharmacy, Duquesne University, Pittsburgh, PA, United States; ^2^Division of Medicinal Chemistry, School of Pharmacy, Duquesne University, Pittsburgh, PA, United States; ^3^Department of Medicine-Section of Hematology and Medical Oncology, Tulane University, New Orleans, LA, United States

**Keywords:** MET, EMT, phenotypic switch, mesenchymal, TNBC, MAPK

## Abstract

Epithelial to mesenchymal transition (EMT) is a cellular program that converts non-motile epithelial cells into invasive mesenchymal cells. EMT is implicated in cancer metastasis, chemo-resistance, cancer progression, and generation of cancer stem cells (CSCs). Inducing mesenchymal to epithelial transition (MET), the reverse phenomenon of EMT, is proposed as a novel strategy to target triple negative and tamoxifen-resistant breast cancer. Triple negative breast cancer (TNBC) is characterized by the loss of hormone receptors, a highly invasive mesenchymal phenotype, and a lack of targeted therapy. Estrogen receptor-positive breast cancer can be targeted by tamoxifen, an ER antagonist. However, these cells undergo EMT over the course of treatment and develop resistance. Thus, there is an urgent need to develop therapeutic interventions to target these aggressive cancers. In this study, we examined the role of novel diphenylamine analogs in converting the mesenchymal phenotype of MDA-MB-231 TNBC cells to a lesser aggressive epithelial phenotype. Using analog-based drug design, a series of diphenylamine analogs were synthesized and initially evaluated for their effect on E-cadherin protein expression and changes incell morphology, which was quantified by measuring the spindle index (SI) value. Selected compound **1** from this series increases the expression of E-cadherin, a primary marker for epithelial cells, and decreases the mesenchymal markers SOX2, ZEB1, Snail, and vimentin. The increase in epithelial markers and the decrease in mesenchymal markers are consistent with a phenotypic switch from spindle-like morphology to cobblestone-like morphology. Furthermore, Compound **1** decreases spheroid viability, cell migration, and cell proliferation in triple negative BT-549 and tamoxifen-resistant MCF-7 breast cancer cells.

## Introduction

Breast cancer is the most common cancer in women^1^. Approximately 10-20% of breast cancers are triple-negative breast cancer (TNBC) (1). TNBC cells do not express estrogen, progesterone, or HER2 (human epidermal growth factor receptor 2) receptors, do not respond to traditional endocrine therapy, and require a more aggressive chemotherapeutic strategy (2). Unfortunately, only 50% of patients with TNBC respond to chemotherapy (3). After decades of drug-discovery efforts, there is no preferred drug approved for the treatment of TNBC (4). While estrogen positive breast cancer can be targeted with ER antagonists such as tamoxifen, about 30% patients develop resistance to the therapy and the recurrent tumors require more rigorous treatment (5). Therefore, exploration of new therapies is warranted in such aggressive cancers.

Epithelial to mesenchymal transition (EMT) is a reversible cellular process where cobblestone-like, less motile differentiated epithelial cells undergo morphological changes to acquire a more mobile invasive mesenchymal-like phenotype (6–8). Data from two independent studies indicate that reduction in E-cadherin expression is a critical event in the metastases and recurrence of aggressive lobular breast carcinomas (9, 10). Decreased expression of E-cadherin is mediated by mesenchymal-associated transcription factors including Snail, Slug, and zinc finger E-box binding homeobox1 (ZEB1) (11, 12). Even though less is known about the reversion from a mesenchymal-like cell type to an epithelial-like phenotype and re-expression of E-cadherin (13), recent studies highlight the potential of MET as an effective anti-cancer strategy (14–16).

Increasing evidence suggests that EMT plays an instrumental role in breast cancer metastasis (17–20). Considering the central role of EMT in cancer progression, targeting EMT represents an attractive approach to treat cancer (21). Additionally, EMT is proposed to play a central role in the development of cancer stem-like cells (22, 23) and chemo-resistance (24). Most invasive cancers, including triple negative (TNBC) and tamoxifen resistant (TAMR) breast cancers have a mesenchymal phenotype. Induction of the MET program by small molecules therefore represents a practical and viable approach for treating these cancers (14–16).

While most small-molecule kinase inhibitors target the ATP-binding pocket, several novel diphenylamine derivatives were synthesized to target an allosteric site of MEK5 (25). Targeting allosteric pockets of kinases may offer reduced resistance and greater kinase selectivity (26). Compound **1** was identified to target the EMT axis in MDA-MB-231 cells and attenuate the tumorigenic characteristics of cancer cells in several *in vitro* assays. The highly invasive MDA-MB-231 cell line (TNBC) was utilized, as it consists of more than 90% of high CD44^+^/CD24^−^/low stem cells (27), and has high expression of mesenchymal markers including vimentin, Snail, Slug, and cadherin 11. Structural variations of the diphenylamine structure were conducted with the goal of determining if the observed MET arose from discrete chemical/physical properties tractable to lead optimization vs. bulk chemical properties. We proposed two strategies to quantify the activity of diphenylamine derivatives for inducing mesenchymal to epithelial transition in these cells: (i) upregulation of the epithelial marker E-cadherin and (ii) phenotypic switch from mesenchymal to epithelial after treatment with structural analogs of compound **1** as indicated by reduction in spindle index. To our knowledge, this is the first time a series of novel diphenylamine analogs are shown to induce MET in TNBC. Compounds that induce E-cadherin protein expression and alter the mesenchymal cell morphology to epithelial, as indicated by the reduction in the spindle index, are termed as “MET-activators.” Given the structures of the active compounds (two aromatic rings separated by a heteroatom), a survey of established drugs and endogenous compounds was conducted to see if prior compounds with structural similarities possessed similar ability to induce MET. Tolfenamic acid and thyroid hormones also contain two aromatic rings connected by a single heteroatom and they therefore were also evaluated for MET activity (Supplemental Figure 1). Sulindac and Meloxicam (NSAIDs) were chosen because they possess anti-cancer activities by inhibiting EMT (28, 29).

The lead molecule, analog **1**, was further tested in TNBC cell lines (MDA-MB-231 and BT-549) and tamoxifen-resistant (TAMR) MCF-7 breast cancer cell lines and found to decrease spheroid formation, cell migration, and cell proliferation *in vitro*. Additionally, we have previously shown that compound **1**, inhibits TNBC tumorigenesis *in vivo* (25).

## Methods and Materials

### Cell Culture and Reagents

MDA-MB-231, BT-549, and MCF-7 cells were obtained from American Type Culture Collection (ATCC). MDA-MB-231 cells were maintained in Dulbecco's Modified Eagle Medium and Ham F-12 (1:1), BT-549 and MCF-7 cells were maintained in RPMI-1640 medium supplemented with 5% FBS (Gibco) and 0.5% Pen Strep (Gibco) in a humified atmosphere containing 5% CO_2_ at 37°C.

### Generation of Tamoxifen-Resistant MCF-7 Cell Line

The MCF-7 cells were cultured in phenol red-free RPMI-1640 media and 5% charcoal-stripped FBS (to remove endogenously expressed protein growth factors present in the media) in the presence of DMSO or (*Z*)-4-Hydroxytamoxifen (4-OHT) (Sigma-Aldrich Cat. No. H7904) at a concentration of 0.1 μM for 6 months.

### Crystal Violet Staining

The cells were seeded at a density of 50,000 cells/well in a 12-well plate, treated with 1 μM concentration of compounds after 24 h, and allowed to grow for 5 days after treatment. At the end-point, media was aspirated and cells were washed with PBS. The cells were then fixed with 100 μl 4% paraformaldehyde per well for 15 min. The cells were washed once with PBS and stained with 50 μl crystal violet per well for 15 min. The cells were washed with 100 μl PBS three times. Pictures were taken using EVOS™ FL inverted microscope (Life Technologies) under 10X magnification.

### Spindle Index Calculation

Spindle indices (SI) of individual cells were calculated from at least 200 cells per treatment from a minimum of three images as the ratio of length (l) to width (w); SI = l/w of each cell. Cells with SI < 3 were considered as epithelial. The percentage of cells <3 were calculated as the ratio of the number of cells with spindle index <3 to the total number of cells per image. The method was adopted from reference (30). Length and width were measured using the Image J software, U. S. National Institutes of Health, Bethesda, Maryland, USA.

### Western Blot Analysis

Cells were seeded in 12-well plates at a seeding density of 50,000 cells per well in 1 mL full media and treated with compounds after 24 h at a 1 μM concentration. The cells were lysed in 1X ice-cold RIPA buffer (Cell Signaling Technology Cat. No. 9803S) and protease inhibitor cocktail (Cell Signaling Technologies Cat. No. 5871S). The proteins were resolved using 8% SDS polyacrylamide gel electrophoresis and transferred to nitrocellulose membranes (LI-COR Biosciences; Lincoln, NE). The membranes were blocked for 1 h at room temperature and incubated at 4°C overnight with E-cadherin, ZEB1, Snail, Slug, primary antibodies, Cell Signaling EMT Antibody Sampler Kit (1:1000, Catalog No. 9782S), SOX2 antibody (1:1000, Millipore, Cat. No. AB5603S), and α-tubulin (1:10000; Cell Signaling Technology, Cat. No. 2144). The antibodies were diluted in casein blocking buffer (LI-COR Biosciences). The membranes were washed thrice with wash buffer (PBS 1X, Tween 0.02%). The membranes were incubated with goat anti-rabbit (1:10000, Invitrogen) and goat anti-mouse (1:10000, Invitrogen) secondary antibodies for 1 h. Membranes were washed thrice with the PBS-tween wash buffer and scanned on an LI-COR's Odyssey CLx Imager at 700 nm (goat anti-rabbit) and 800 nm (goat anti-mouse). The blots were quantified with Image Studio Software. Results are represented as protein expression of treated group vs. DMSO control as ± SEM of experiments repeated at least three times.

### Colony Formation Assay

Colony formation was performed using a Soft Agar Colony Formation assay (Cell Biolabs; CBA-130) manufacturer's protocol. Base agar layer was added first. MDA-MB-231 cells were cultured in 5% FBS growth media and 1.2% agar solution at a seeding density of 5,000 cells/well. DMSO or compound **1** at 0.1, 1, and 10 μM concentrations were added on the top of cell layer. The colonies were seeded to grow for 7 days. The agar layer was then solubilized, colonies were lysed, and stained with CyQuant dye. Florescence was measured on VICTOR3 1420 Perkin Elmer multi-label counter at 485 nm.

### Spheroid Culture

Cells were cultured in 96-well low attachment plates (Corning Cat. No. 4520) at a seeding density of 5,000 cells/ well. DMSO or compound **1** at 0.1, 1, and 10 μM concentrations was added after 24 h. Pictures were taken using the EVOS™ FL inverted microscope (Life Technologies) under 4X magnification. At the time of treatment and after 7 days from the time of treatment. After 7 days of treatment, 10 μl of Reliablue viability reagent (ATCC® 30-1014™) was added to each well. The plates were incubated for 3 h in the incubator at 37°C. The absorbance was measured at a wavelength of 570 nm using Wallac 1420 software on a Perkin Elmer 1640 multi-label counter. Results are represented as spheroid viability normalized to DMSO control ± SEM of triplicate experiments.

### Scratch Assay

Cell migration was assessed using a scratch assay after treatment with DMSO or compound **1** at 0.1, 1, and 10 μM concentrations for 72 h. Cells were seeded in 12-well plates at a seeding density of 50,000 cells/ well in 1 ml full media in the presence of 5% FBS. Scratches were made after 48 h of the treatment. Images were taken at the time of scratch and after 24 h from the time of scratch. The current scheme of treatment was employed to observe the effect of the treatment on cell migration at a longer time-point. The images and scheme of treatment are included in the Supplemental Figure 3. The closure rates were calculated as Wound closure = (border width at 24 h-border width at 0 h) X 100. Results are represented as wound closure normalized to DMSO control ± SEM of triplicate experiments repeated three times.

### Immunofluorescence Assay

Cells were plated at a density of 5,000 cells/well in a 96-well plate and treated with increasing concentrations of compound **1** (0.1, 1, and 10 μM). After 72 h of treatment, cells were fixed with 4% paraformaldehyde for 15 min and blocked for 1 h with 0.3% Triton-X solution. The primary antibody rabbit Ki67 antibody (1:1000, Cell Signaling Technology) was applied to stain the proliferating cells, and the cells were incubated overnight at 4°C. The cells were subsequently washed three times, incubated for 1 h with goat anti-mouse Alexa Flour 488 nm and goat anti-Rabbit Alexa Flour 555 nm (1:1000, Invitrogen), and counterstained with Hoechst (Fisher) to visualize the nucleus. The plate was imaged with the EVOS™ FL inverted microscope (Life Technologies) under 10X magnification. The proliferative index was calculated as the ratio of number of Ki67^+^ cells to the number of Hoechst^+^ cells. Results are represented as cell proliferation normalized to DMSO control ± SEM of triplicate experiments repeated three times.

### Cell Viability Assay

MTT (3-(4,5-dimethylthiazol-2-yl)-2,5-diphenyltetrazolium bromide) assay was performed to determine cell viability. Cells were seeded at a density of 5,000 per well in 96-well plates containing 90 μl of full media for 24 h and then treated with 1 μM compound for 72 h. After treatment, 10 μL of MTT (Acros, Cat. No. 298-93-1) solution (5 mg/ml in phosphate-buffered saline, PBS) was added to each well and incubated at 37°C for 3 h. After removal of the MTT solution, 100 μl of DMSO was added to the wells for 10 min under agitation to dissolve the formazan crystals. Absorbance was measured at a wavelength of 570 nm using Wallac 1420 software on a Perkin Elmer 1640 multilabel counter. Results are represented as cell viability normalized to DMSO control ± SEM of triplicate experiments repeated three times (Supplemental Figure 4).

### Statistical Analyses

Two-tailed Student's unpaired *t*-test was used in statistical analyses that involved a comparison between treated (diphenylamine derivatives) and control (DMSO) groups (fold change, E-cadherin and spindle index). If *p*-values were below 0.05, differences were considered significant. Data represent ± SEM of at least three independent experiments. One-way analysis of variance (ANOVA) with Bonferroni *post-hoc* correction was used to examine concentration-dependent effect of compound **1** on cell viability, proliferation, spheroid viability, and cell motility. Two-tailed Pearson correlation analysis and linear regression was used for correlation studies. Statistical analyses were performed using GraphPad Prism version 7.03 for Windows, GraphPad Software, La Jolla California USA.

## Results

### Compound 1 Induces MET and Decreases Colony Formation, Cell Migration, Spheroid Formation, and Cell Proliferation in MDA-MB-231 Cells

**Figure 1:**
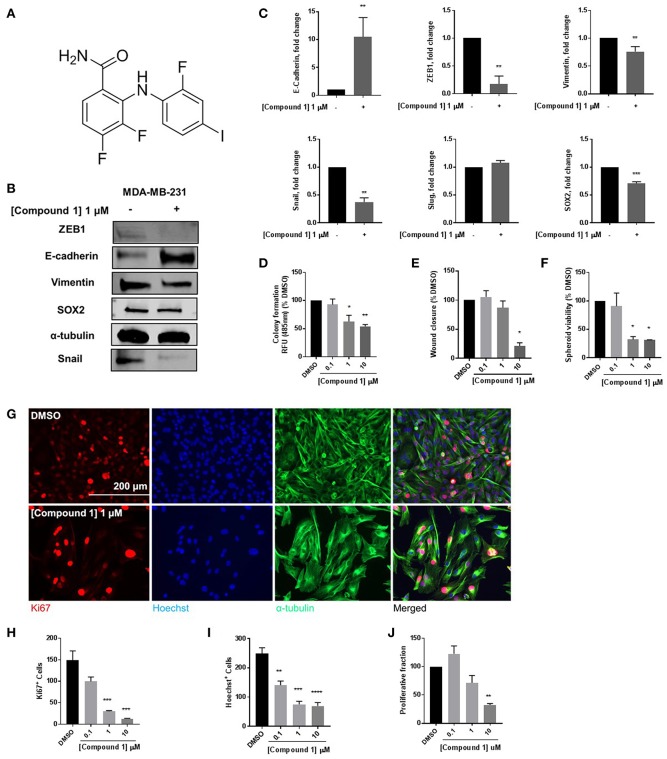
Compound 1 induces MET and decreases colony formation, cell migration, and spheroid formation in MDA-MB-231 cells. **(A)** Structure of compound **1**. **(B)** Western blot of epithelial marker E-cadherin, mesenchymal markers Vimentin, Snail, and ZEB1, and stemness marker SOX2 in MDA-MB-231 cells. **(C)** Quantification of protein expression of E-cadherin, ZEB1, vimentin, Snail, Slug, and SOX2. Data represent ± SEM **p < 0.01; ***p < 0.001; vs. DMSO control group determined by two-tailed Student's *t*-test. **(D)** Compound **1** inhibited MDA-MB-231 colony formation after 14 days of treatment in a concentration-dependent manner. Data represent ± SEM of experiments run in triplicate, *p < 0.05; **p < 0.01 vs. DMSO control group determined by one-way ANOVA with the Bonferroni *post hoc* test. **(E)** Wound closure was measured as a percentage of untreated DMSO control group (missing compound 1 at various conc here). *p < 0.05 vs. DMSO control group determined by one-way ANOVA with the Bonferroni *post hoc* test (MDA-MB-231 cells). **(F)** Quantification of the spheroid viability in MDA-MB-231 cells, values indicate ± SEM of three experiments run in triplicate. *p < 0.05 vs. control group determined by one-way ANOVA with the Bonferroni *post hoc* test in MDA-MB-231 cells. **(G)** Immunofluorescence staining of Ki67, Hoechst, and α-tubulin in MDA-MB-231 cells treated with DMSO or compound **1** (1 μM), for 72 h, scale bar 200 μm. **(H)** Ki67^+^ cells decreased with increasing concentrations of compound **1**. **(I)** Decrease in Hoechst^+^ cells with increasing concentrations (0.1, 1, 10 μM) of compound **1**. **(J)** The proliferative fraction calculated as the ratio of Ki67^+^ cells to Hoechst^+^ cells at increasing concentration. Data represent ± SEM of three different experiments. **p < 0.01; ***p < 0.001 vs. control group determined by one-way ANOVA with the Bonferroni *post hoc* test.

At 1 μM concentration, Compound **1** (Figure 1A) increases E-cadherin protein expression vs. non-treated cells by a factor of 10 and decreases expression of mesenchymal markers ZEB1, Snail, and vimentin in MDA-MB-231 cells (Figures 1B,C). Moreover, compound **1** decreases the protein expression of stem cell marker SOX2 (Figure 1C) and colony formation (Figure 1D). The effects of compound **1** on MET are consistent with concentration-dependent reduction in cell migration (Figure 1E), and spheroid formation (Figure 1F) in MDA-MB-231 cells, which are important assays for studying MET. Additionally, compound **1** significantly inhibits cell proliferation, determined by immunofluorescence staining for Ki67 and Hoechst (Figures 1G–J) in MDA-MB-231 cells.

### Identification of Diphenylamine Scaffold as an Activator of MET

**Figure 2:**
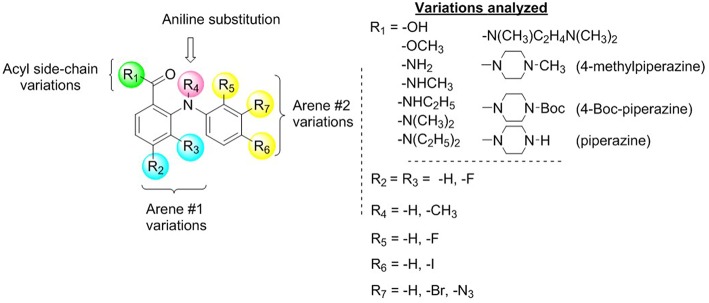
Identification of diphenylamine scaffold as an activator of MET.

**Figure 3:**
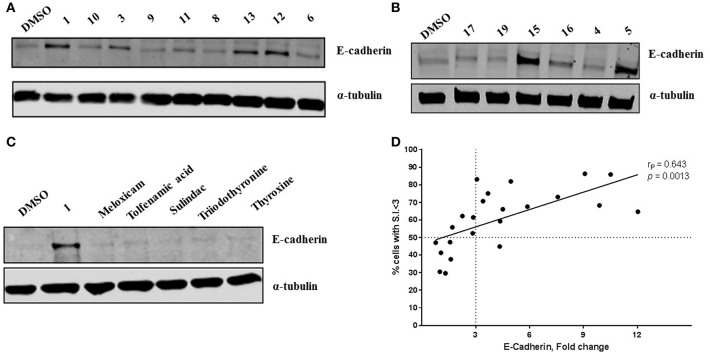
Novel diphenylamine analogs induce E-Cadherin protein expression in MDA-MB-231 cells **(A,B)**. Western blot images of MDA-MB-231 cells treated with novel diphenylamine compounds at 1 μM concentration for 5 days. E-Cadherin protein expression is normalized to α-tubulin and the data are summarized in Table 1. **(C)** E-Cadherin expression after treatment with NSAIDs and thyroid hormones in MDA-MB-231 cells (5 days treatment at 1 μM concentration). **(D)** Increase in E-cadherin expression significantly correlates with the percentage of cells that exhibit a phenotypic switch. A positive correlation between E-Cadherin protein expression, compared to DMSO and an increase in percentage of cells with S.I < 3 was observed. Data was analyzed by two-tailed Pearson correlation. The correlation coefficient (rP) and significance (*p*-value) are indicated on the scatter plot.

Compound **1** (Figure 1A) increased the expression of epithelial marker E-cadherin and induced a phenotypic switch from spindle-like to cobblestone-like morphology. Collectively, these observations are consistent with a MET induction or EMT reversal. A structure-activity correlation was conducted to identify the atoms and functional groups essential for MET induction and to identify the most active MET inducers from our diphenylamine drug library. The compounds were tested on MDA-MB-231 cells and were initially analyzed for an increase in E-cadherin expression and change in morphology from mesenchymal to epithelial phenotype as indicated by a decrease in the spindle index. As shown in Figure 2, four locations of structural variations for the structure-activity relationship were chosen, and various substitutions were made. The increase in E-cadherin expression by diphenylamine analogs in MDA-MB-231 cells is shown in Figures 3A,B. The structural variations along with the E-cadherin fold change mean, SI values (30), and % cells with SI < 3 are summarized in Table 1.

1. Acyl side chain substitution (R_1_)
2. Arene 1 variations (R_2_ and R_3_)
3. Aniline “NH” hydrogen (R_4_)
4. Arene 2 variations (R_5_, R_6_, and R_7_)

**Table 1:** Effect of diphenylamine derivatives on E-Cadherin, mean spindle index, and % cells with SI<3 in MDA-MB-231 cells.

**#**	**Side chain R_**1**_**	**Arene #1**			**Arene #2**			**E-Cadherin (Fold change ± S.E.M.)^a^**	***p*-value**	**S.I. ± S.E.M.^b^**	**% cells with SI < 3 ± S.E.M.^b^**
		**R_**2**_**	**R_**3**_**	**R_**4**_**	**R_**5**_**	**R_**6**_**	**R7**				
**1**	-NH_2_	-F	-F	-H	-F	-I	-H	10.5 ± 3.5	0.012	2.2 ± 0.1	85.9 ± 3.4
**2**	-NHMe	-F	-F	-H	-F	-I	-H	12 ± 4.6	0.002	2.7 ± 0.2	64.6 ± 0.4
**3**	-NHEt	-F	-F	-H	-F	-I	-H	4.9 ± 1.9	0.001	2.5 ± 0.4	81.9 ± 7.8
**4**	-N(C_2_H_5_)_2_	-F	-F	-H	-F	-I	-H	6.1 ± 2.1	<0.0001	2.6 ± 0.2	67.5 ± 6.2
**5**	-N(CH_3_)_2_	-F	-F	-H	-F	-I	-H	7.6 ± 0.5	<0.0001	2.6 ± 0.2	73.1 ± 5.5
**6**	-OMe	-F	-F	-H	-F	-I	-H	2.3 ± 0.7	0.015	2.9 ± 0.1	62.2 ± 2.4
**7**	-OH	-F	-F	-H	-F	-I	-H	4.5 ± 2.05	0.009	2.8 ± 0.1	66.1 ± 6.4
**8**	-NH_2_	-H	-H	-H	-F	-I	-H	2.9 ± 1.4	0.062	3.3 ± 0.2	61.5 ± 5.1
**9**	-NH_2_	-F	-F	-CH_3_	-F	-I	-H	2.8 ± 1.1	0.008	3.3 ± 0.4	52.4 ± 12
**10**	-NH_2_	-F	-F	-H	-H	-H	-H	1.3 ± 0.1	0.002	3.9 ± 0.3	29.7 ± 1.1
**11**	-NH_2_	-F	-F	-H	-F	-H	-H	4.3 ± 1.5	0.004	3.6 ± 0.1	44.9 ± 0.3
**12**	-NH_2_	-F	-F	-H	-H	-I	-H	3.1 ± 0.6	<0.0001	2.4 ± 0.1	83.1 ± 1.8
**13**	4-methylpiperazine	-F	-F	-H	-F	-I	-H	3.4 ± 0.8	<0.0001	2.8 ± 0.1	70.7 ± 2.1
**14**	4-methylpiperazine	-F	-F	-H	-F	-H	-H	1.1 ± 0.2	0.550	3.5 ± 0.1	41.3 ± 4.7
**15**	-piperazine	-F	-F	-H	-F	-I	-H	9.1 ± 2.2	<0.0001	1.9 ± 0.2	86.3 ± 7.7
**16**	4-Boc-piperazine	-F	-F	-H	-F	-I	-H	9.9 ± 4.1	<0.0005	2.8 ± 0.1	68.3 ± 4.7
**17**	-N(CH_3_)C_2_H_4_N(CH_3_)_2_	-F	-F	-H	-F	-I	-H	4.3 ± 3.3	0.048	3 ± 0.2	59.3 ± 6.1
**18**	4-methylpiperazine	-F	-F	-H	-H	-H	-H	1.7 ± 0.47	0.0009	3.6 ± 0.1	55.8 ± 5.2
**19**	4-methylpiperazine	-F	-F	-H	-H	-H	-N_3_	1.6 ± 0.90	0.2786	3.5 ± 0.2	47.4 ±2.9
**20**	-NHEt	-F	-F	-H	-H	-H	-N_3_	0.77 ± 0.01	<0.0001	3.4 ± 0.2	47.1 ± 6.7
**21**	4-methylpiperazine	-F	-F	-H	-H	-H	-Br	1.6 ± 0.88	0.2394	3.8 ± 0.2	37.6 ± 5.5
	DMSO							1		4.2 ± 0.4	30.5 ± 4.8

a *MDA-MB-231 cells were treated compounds at 1 μM concentration for 5 days. Data represent mean ± SEM, unpaired two-tailed Student's t-test (n = 3–7). E-Cadherin was normalized to α-tubulin, fold change is compared to DMSO. One-way ANOVA with Bonferroni post-hoc comparison analysis where the compounds were compared to the DMSO control and to each other; compounds **1** and **2** were found to be statistically significant compared to the DMSO control group (P < 0.05). We missed significance across groups because there was large difference between the minimum and the maximum effect produced by the different compounds. Therefore, we switched to performing t-test and compared each compound individually to the DMSO control group*.

b *Data represent the ± SEM of three different experiments determined by unpaired two-tailed Student's t-test (n = 3)*.

At R_1_, E-cadherin expression order for amide variations was NHCH_3_ (**2**) > NH_2_ (**1**) > 4-Boc-piperazine (**16**) > piperazine (**15**) > N(CH_3_)_2_ (**5**) > N(C_2_H_5_)_2_ (**4**) > NHC_2_H_5_ (**3**) > N(CH_3_)C_2_H_4_N(CH_3_)_2_ (**17**) > 4-methylpiperazine (**13**; Table 1). E-cadherin expression was significantly upregulated after treatment with compounds **1**, **2**, **15**, and **16**, causing a >9-fold increase compared to DMSO. The 4-methyl piperazinyl derivative **13** was 3-fold less active when compared to parent compound **1**. Removal of the 4-methyl group leads to 3-fold improvement in activity (compound **13** vs. **15**). Moreover, the 4-Boc protected derivative **16** was more 3-fold more active than **13**. Compounds **15** and **16**, were equipotent to the parent compound **1**. Ester **6** was about 5-fold less potent, and the acid (**7**) was 2-fold less active when compared to **1**. Both neutral amides (**1-5**, **16**) and basic side chain amides (**15**) were active in promoting MET consistent with significant functional group tolerance attached to the amide group.

Replacement of the R_2_ and R_3_ fluoro atoms with hydrogens led to a >3-fold decrease in the E-cadherin expression (**1** vs. **8**). Similarly, replacing the R_4_ hydrogen with a methyl group leads to a >3-fold reduction in the E-cadherin levels (**1** vs. **9**).

The Arene 2 substitution (R_5_, R_6_, and R_7_) showed significant change with atom replacement. Removal of either the iodo atom (**11**) at R_6_ or the fluoro atom (**12**) at R_5_results in a 3-fold decrease in E-cadherin expression as compared to **1**. Furthermore, removal of both the groups seen in compound **10**, E-cadherin was undetectable in our assays. These data suggest that the fluorine atom at R_5_ and the iodine atom at R_6_ are essential for MET induction. The analogs with substitutions at the R_7_ position were found to inactive (**19**–**21**). As mentioned before, since tolfenamic acid and thyroid hormones bear's structural resemblance to compound **1**; sulindac and meloxicam inhibits EMT, a focused survey of these compounds was conducted for induction of E-cadherin expression. No increase in E-cadherin expression (Figure 3C) was noted for these compounds.

### Increase in E-Cadherin Expression Correlates With a Phenotypic Switch in MDA-MB-231 Cells

Morphological switch from a spindle-like phenotype to a cobblestone-like phenotype is key characteristic of cells that undergo MET. Crystal violet staining was performed after 5 days of treatment to examine cell morphology. MDA-MB-231 cells undergo a phenotypic switch from mesenchymal to epithelial after treatment with diphenylamine derivatives (Supplemental Data Sheet 1, Figure 2). To quantitatively measure this MET, the spindle index (SI) was calculated. The compounds that increased E-cadherin protein expression significantly also altered the morphology of MDA-MB-231 cells from mesenchymal to epithelial; this was consistent with a significant reduction in the spindle index value. Cells with a SI < 3 were considered epithelial. Compounds that increased E-cadherin by at least 3-fold and that displayed >50% of the cells with a SI < 3 were described as MET activators (Figure 3D). The overall results show a direct correlation between the increase in E-cadherin with the decrease in spindle index (Figure 3D). The most potent MET activators from this series were analogs **1**, **2**, **15**, and **16** (Table 1, and Figure 3D) inducing a more than 9-fold increase in E-cadherin as compared to DMSO (vehicle) and with a SI < 3. Combining increase in the E-cadherin expression with decrease in the SI; analogs **1** and **15** showed the best profile in increasing E-cadherin (10.5 and 9.1 fold increase respectively vs. 1 with DMSO), decreasing SI (2.2 and 1.9 respectively vs. 4.2 with DMSO) along with highest percentage of cells with SI < 3 (85.9 and 86.3% respectively vs. 30.5% with DMSO) and they were further tested in spheroid viability assay. Analog **18** was used as a negative control.

### Effect of Compounds 18, 15, and 1 on Spheroid Viability

**Figure 4:**
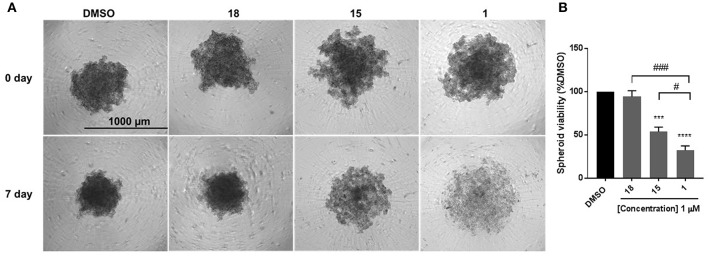
Compound 1 and 15 decrease spheroid viability in MDA-MB-231 cells. **(A)** Spheroid formation in MDA-MB-231 cells after treatment with DMSO or compounds **18**, **15**, and **1** for 7 days of treatment. **(B)** Quantification of the spheroid viability in MDA-MB-231 cells, values indicate ± SEM of three experiments run in triplicate. ***p < 0.001, ****p < 0.0001 vs. control group, ^*###*^*p* < 0.001 compound **1** vs. compound **18** ^#^*p* < 0.05 compound **1** vs. compound **15** determined by one-way ANOVA with the Bonferroni *post hoc* test in MDA-MB-231 cells.

EMT is associated with resistance to anchorage-independent death “anoikis,” and metastasis (31). To evaluate the effect of diphenylamines on anoikis-resistant cells, MDA-MB-231 cells were grown in ultra-low-attachment plates to form spheroids. Compound **1** significantly decreased spheroid viability compared to DMSO, compound **18**, and compound **15** (Figures 4A,B). The major conclusion from this experiment was that compound **1** was the best in-series. Hence its effects on spheroid formation, cell migration, and cell proliferation were further evaluated in BT-549 and TAMR MCF-7 cells, which also have a mesenchymal phenotype.

### Compound 1 Inhibits Spheroid Formation and Cell Migration in BT-549 and TAMR MCF-7 Cells

**Figure 5:**
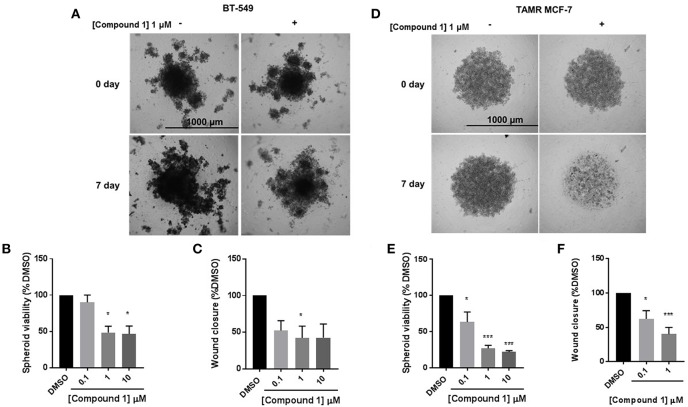
Compound 1 inhibits spheroid viability and cell migration in BT-549 and TAMR MCF-7 cells. **(A)** Spheroid formation in BT-549 cells after treatment with DMSO or compound **1** at 1 μM concentration for 7 days, scale bar 1,000 μm. **(B)** Quantification of the spheroid viability in BT-549 cells after treatment with compound **1** at increasing concentrations (0.1, 1, 10 μM) after 7 days of treatment, data represents ± SEM *p < 0.05; ***p < 0.001 vs. control group determined by one-way ANOVA with the Bonferroni *post hoc* test in BT-549 cells. **(C)** Wound closure was measured after treatment with increasing concentrations (0.1, 1, 10 μM) of compound **1**. The data is presented as a percentage of untreated DMSO control group. *p < 0.05 vs. control group determined by one-way ANOVA with the Bonferroni *post hoc* test (BT-549 cells). **(D)** Spheroid formation in TAMR MCF-7 after treatment with DMSO or compound **1** at 1 μM concentration for 7 days, cells scale bar 1,000 μm. **(E)** Quantification of the spheroid viability after treatment with compound **1** at increasing concentrations (0.1, 1, 10 μM) after 7 days of treatment in TAMR MCF-7 cells, values indicate ± SEM of three experiments run in triplicate. *p < 0.05 vs. control group determined by one-way ANOVA with the Bonferroni *post hoc* test in TAMR MCF-7 cells. **(F)** Wound closure was measured after treatment with increasing concentrations (0.1, 1, 10 μM) of compound **1**. The data is presented as a percentage of untreated DMSO control group. *p < 0.05; ***p < 0.001 vs. control group determined by one-way ANOVA with the Bonferroni *post hoc* test (TAMR MCF-7 cells).

The cells were treated with 0.1, 1, and 10 μM concentrations of compound **1**. Treatment with compound **1** led to a concentration-dependent reduction in spheroid viability after 7 days in BT-549 cells (Figures 5A,B) and TAMR MCF-7 cells (Figures 5D,E). At 1 μM concentration, compound **1** treatment decreased 51.55 and 72.91% spheroid viability in BT-549 (Figure 5B) and TAMR MCF-7 cells (Figure 5E), respectively. Additionally, compound **1** produced a concentration-dependent decrease in cell migration in both BT-549 (Figure 5C) and TAMR MCF-7 cells (Figure 5F).

### Compound 1 Decreases Cell Proliferation in BT-549 and TAMR-MCF-7 Cells

**Figure 6:**
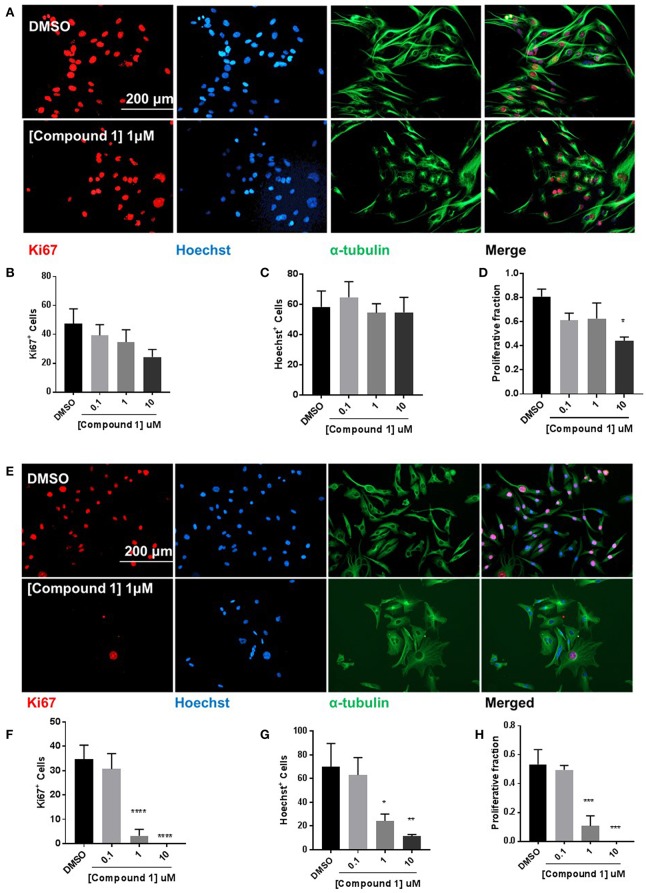
Compound 1 inhibits cell proliferation in BT-549 and TAMR MCF-7 cells. **(A)** Immunofluorescence staining of Ki67, Hoechst, and α-tubulin in BT-549 cells treated with DMSO or compound **1** (1 μM), for 72 h, scale bar 200 μm. **(B)** Ki67^+^ cells decreased with increasing concentrations (0.1, 1, 10 μM) of compound **1**. **(C)** Decrease in Hoechst^+^ cells with increasing concentrations (0.1, 1, 10 μM) of compound **1**. **(D)** The proliferative fraction calculated as the ratio of Ki67^+^ cells to Hoechst^+^ cells at increasing concentrations (0.1, 1, 10 μM) of compound **1**. Data represent ± SEM of three different experiments. *p < 0.05 vs. control group determined by one-way ANOVA with the Bonferroni *post hoc* test. **(E)** Immunofluorescence staining of Ki67, Hoechst, and α-tubulin in TAMR MCF-7 cells treated with DMSO or compound **1** (1 μM), for 72 h, scale bar 200 μm. **(F)** Ki67^+^ cells decreased with increasing concentrations (0.1, 1, 10 μM) of compound **1**. **(G)** Decrease in Hoechst^+^ cells with increasing concentrations (0.1, 1, 10 μM) of compound **1**. (TAMR MCF-7 cells) **(H)** The proliferative fraction calculated as the ratio of Ki67^+^ cells to Hoechst^+^ cells at increasing concentrations (0.1, 1, 10 μM) of compound **1** (TAMR MCF-7 cells). Data represent ± SEM of three different experiments. *p < 0.05; **p < 0.01; ***p < 0.001; ****p < 0.0001 vs. control group determined by one-way ANOVA with the Bonferroni *post hoc* test.

Compound **1** produced a concentration-dependent decrease in proliferation in BT-549 cells (Figure 6A). A decrease in the number of Ki67^+^ (Figure 6B) and Hoechst^+^ (Figure 6C) was observed. The proliferative fraction was measured as the ratio of the number of Ki67^+^ to the number of Hoechst^+^ cells. There was a 67.9% reduction in cell proliferation at 10 μM concentration (Figure 6D). The anti-proliferative effect was more pronounced in the TAMR MCF-7 cells (Figure 6E) where we observed a significant decrease in the Ki67^+^ (Figure 6F) and Hoechst^+^ (Figure 6G) cell numbers. At 1 μM, there was 79.7% decrease in the proliferative fraction (Figure 6H).

## Discussion

Based on the discovery that compound **1** induced MET in MDA-MB-231 cells and reduced stemness, migration, and spheroid formation, we hypothesized that by performing analog-based drug design we would be able to identify the atoms or functional groups essential for the activity. This information would then be utilized in the design of second generation compounds to further improve MET activity. Our initial findings suggest that at R_1_ position amides (primary, secondary, and tertiary) are better compared to esters (**6**) or acid (**7**). The R_1_ position also showed tolerance toward charge and steric bulk. This offers a unique opportunity for attachment of a probe to deduce the molecular target of diphenylamines toward activating MET. At arene #1 (R_2_ and R_3_) the removal of both the fluoro groups reduces the E-cadherin expression by 3.5 fold (**8** vs. **1**). We postulate that the electron-withdrawing fluorine atoms either polarize the arene 1 ring potentiating its interactions with its biological target (π-π stacking interactions and/or ion-dipole interactions) or the fluorine atom may act as hydrogen bond acceptor (HBA) (32). At R_4_ position the replacement of the hydrogen atom (**1**) with a methyl group (**9**) reduces the E-cadherin expression by 4-fold. We hypothesize that the R_4_ hydrogen participates in an intramolecular hydrogen bond with the carbonyl group attached to R_1_, forming a conformation favoring MET. Since the presence of the N-methyl group of analog **9** disrupts the intramolecular hydrogen bond we see a corresponding decrease in the E-cadherin expression (Supplemental Figure 5). By removing fluorine at R_5_ and iodine at R_6_ we observed complete abolishment in E-cadherin expression (analog **10** vs. **1**). We postulate that the fluoro group at R_5_ may serve multiple chemical and structural features: a) it decreases the pKa of the R_4_ hydrogen making it a better hydrogen bond donor to facilitate MET induction, and/or b) it increases lipophilicity of the molecule; improving hydrophobic interactions with the biological target(s) (33, 34). The iodo group at R_6_ is a large group, that can undergo hydrophobic interactions and/or can form a halogen bond. At R_7_ position all the three analogs (**19**-**21**) were found to be inactive in inducing MET. An important point to remember here is that these compounds do not have any substitutions at R_5_ and R_6_ position and they did not possess free NH_2_ group at R_1_, which may be a contributing factor in their inability to induce MET. Overall our initial structure-activity correlation suggests that at R_1_ different substitutions are tolerated and hence this position can be exploited toward improving potency or deducing the molecular target of diphenylamimes. Removal/change of substituents at other positions (R_2_-R_7_) are not tolerated and therefore they may represent the minimum pharmacophore required for MET activity.

The diphenylamine analogs were initially synthesized with the goal to selectively target the MEK5/ERK5 pathway. Disruption of actin skeleton via Ras and Src mediated activation of extracellular regulated kinase 1/2 (ERK1/2) and ERK5 is reported, indicating their role in oncogenic transformation and EMT (35). However, the discrete target(s) for MET activation is unknown. As the diphenylamine core has been extensively utilized in the design of MEK1, 2 and 5 inhibitors as type-III inhibitors (allosteric site), it is highly likely that the analogs prepared in this series may be interacting with allosteric sites on MEK1, 2, and 5, or the allosteric sites of other kinases (26, 36). Type-III kinase inhibitors have been developed for other kinases (26) and pseudokinases (37, 38). These type-III binding sites consistently present a three-component pharmacophore: a deep hydrophobic pocket where one or two arenes can interact, a site for an HBA from ammonium (the catalytic site lysine) and a solvent exposed polar binding region near the C-helix. Characterization of the molecular mechanism of action of these diphenylamine analogs inducing MET is underway.

A hypothesis that NSAIDS (tolfenamic acid, sulindac, and meloxicam) and thyroid hormones (triiodothyronine and thyroxine) bearing structural similarity to these compounds could induce MET (by increasing E-cadherin expression) in MDA-MB-231 cells, is not supported by our findings. One reason could be both NSAIDs and thyroid hormones do not contain the minimum pharmacophore required for inducing MET. Also, our diphenylamines may interact different biological targets.

We observed strong correlation between increase in E-cadherin protein expression and the percentage of cells with SI < 3 per treatment, which strengthens the structure-activity relationship. This, also suggests that spindle index measurement could be a reliable quantitative analysis for cells that undergo morphological transitions. The morphology of the cells following treatment with test compounds is included in the Supplemental Figure 2. The compounds were also tested for inhibition of cell viability, since a significant reduction in cell number after treatment with these analogs was observed while being screened for cell morphology (Supplemental Figure 4). The correlation between SI and E-cadherin identified analog **1**, **2**, **15**, and **16** as the most potent MET inducers.

Anchorage-independent cells undergo programmed cell death through a process termed as anoikis. Cancer cells detach from the surrounding cells in the tumor stroma, overcome anoikis to invade blood vessels, and metastasize to other organs. During EMT, downregulation of E-cadherin and upregulation of mesenchymal markers promote the escape of cells from the primary tumors and acquisition of the mesenchymal phenotype is functionally associated with anoikis-resistance and greater spheroid formation ability (31). Hence reduction in spheroid formation is an important characteristic of cells that undergo MET. Cells were cultured in low-attachment plates were examined to determine the effect of compounds **18, 15**, and **1** on spheroid formation. The selection of compounds was made based on the differences in their structures (primary vs. tertiary amides) and their ability to induce MET: low (compound **18)**—high (compounds **1** and **15)**. MDA-MB-231 cells were cultured in low-attachment plates and examined to determine the effect of compounds **18, 15**, and **1** on spheroid formation. Statistical comparison among the three groups revealed that compound **1** was the most effective in inhibiting spheroid growth, hence was selected for characterization in two additional breast cancer cell models, which have a mesenchymal phenotype: TNBC cell line BT-549 and tamoxifen-resistant MCF-7 cell line.

We observed that chronic treatment of MCF-7 cells with 4-Hydroxytamoxifen (4-OHT) activated the EMT program. This was confirmed by observing the morphological change from cobblestone-like to spindle-like and determining E-cadherin protein expression, which was found to be significantly downregulated in TAMR MCF-7 cells compared to the wild type MCF-7 cells (Supplemental Figure 6). The lead diphenylamine molecule (compound **1)** is effective in various functional assays descriptive of MET induction in BT-549 TNBC cells and TAMR MCF-7 cells, which have a mesenchymal phenotype. Compound **1** showed inhibition of spheroid formation in these cell lines. Since EMT triggers increased cell migration and motility, the wound healing assay was conducted. Inhibition of cell motility with compound **1** treatment indicated an attenuation of migratory potential of BT-549 and TAMR MCF-7 cells.

During EMT, upregulation of mesenchymal transcription factors suppresses the expression of proteins important for cell proliferation, resulting in a reduction in cell proliferation (21). Cells that undergo MET re-acquire the proliferative potential to promote metastatic colonization (21). Therefore, we wanted to examine the role of compound **1** on cell proliferation. Compound **1** inhibited the number of Hoechst-positive and Ki67-positive cells, suggesting cytotoxic as well as cytostatic properties of the compound in both the triple negative breast cancer cell lines (MDA-MB-231 and BT-549) and TAMR-MCF-7 cells. We aim to observe long-term effects of compound **1** on MET, cell proliferation, and examine synergistic effects of treating cells in combination with conventional chemotherapy agents in our future experiments.

The current work is an initial description of efforts toward the identification of novel diphenylamine analogs that promote MET. EMT is a crucial process in cancer progression that promotes cell migration and metastases. Despite advances made in the field, there are no inhibitors that can be used clinically to target the mesenchymal phenotype of cancer cells. Through analog-based drug design, we first identified atoms that potentiate MET. This led to the identification of analog **1**, which led to a potent increase in E-cadherin expression and morphological change from mesenchymal to epithelial phenotype. The important challenges to consider for induced MET include potentiated proliferation and metastatic colonization. As a result, it is important to develop drugs that induce MET, inhibit cell migration, and inhibit cell proliferation. The compound **1** obtained from this series therefore represent a very promising lead since it induces MET along with attenuating migratory and proliferative properties of cancer cells.

Further analyses will be performed to identify the mechanism of action of diphenylamine derivatives. Additionally, the structure-activity relationship performed in this series will aid in the design of more potent inducers of MET. These results will be discussed in due course.

## Data Availability

All datasets generated for this study are included in the manuscript and/or the Supplementary Files.

## Author Contributions

AB and MG contributed equally to the writing and execution of this work. All authors contributed to primary research. MB, JC, and PF contributed to the editing and proofreading.

### Conflict of Interest Statement

The authors declare that the research was conducted in the absence of any commercial or financial relationships that could be construed as a potential conflict of interest.

## Footnotes

^1^*Triple-Negative Breast Cancer*. Available online at: https://www.breastcancer.org/symptoms/diagnosis/trip_neg

## Supplementary Material

Supplemental Figure 1 Structural similarity between diphenylamines **(A)**, tolfenamic acid **(B)**, thyroxine **(C)**, and triiodothyronine **(D)**.

Supplemental Figure 2 Change in MDA-MB-231 cell morphology after treatment with diphenylamine derivatives for 5 days at 1 μM, as examined by crystal violet staining.

Supplemental Figure 3 Images of wound closure at the time of treatment and end-point. **(A)** MDA-MB-231 cells, **(B)** BT-549 cells **(C)** TAMR-MCF-7 cells **(D)** Scheme of treatment.

Supplemental Figure 4 Effect of diphenylamine analogs on cell viability in MDA-MB-231 cells. Data represent ± SEM of three different experiments. ^**^*p* < 0.01; ^***^*p* < 0.001 vs. DMSO control group determined by unpaired two-tailed Student's *t*-test (*n* = 3).

Supplemental Figure 5 Effect of intramolecular hydrogen bonding toward inducing MET.

Supplemental Figure 6 **(A)** TAMR MCF-7 cells are mesenchymal in phenotype compared to the wild type MCF-7 cells. **(B)** E-cadherin protein expression is significantly decreased in TAMR MCF-7 cells compared to wildtype MCF-7 cells. ^***^*p* < 0.001 TAMR vs. wild type MCF-7 determined by two-tailed student's *t*-test.

Supplemental Data Sheet 1 Synthesis of diphenylamine analogs.

## References

[1] National Breast Cancer Foundation, Breast Cancer Facts. (2018) www.nationalbreastcancer.org/breastcancerfacts (accessed March 13, 2019).

[2] Anders CK Carey LA. Biology, metastatic patterns, and treatment of patients with triple-negative breast cancer. Clin Breast Cancer. (2009) 9 (Suppl. 2):S73–81. 10.3816/CBC.2009.s.008 19596646 PMC2919761

[3] De Laurentiis M Cianniello D Caputo R Stanzione B Arpino G Cinieri S. Treatment of triple negative breast cancer (TNBC): current options and future perspectives. Cancer Treat Rev. (2010) 36 (Suppl. 3):S80–6. 10.1016/S0305-7372(10)70025-6 21129616

[4] Pal SK Childs BH Pegram M. Triple negative breast cancer: unmet medical needs. Breast Cancer Res Treat. (2011) 125:627–36. 10.1007/s10549-010-1293-1 21161370 PMC3244802

[5] Chang M. Tamoxifen resistance in breast cancer. Biomol Therap. (2012) 20:256–67. 10.4062/biomolther.2012.20.3.256 24130921 PMC3794521

[6] Davis FM Stewart TA Thompson EW Monteith GR. Targeting EMT in cancer: opportunities for pharmacological intervention. Trends Pharmacol Sci. (2014) 35:479–88. 10.1016/j.tips.2014.06.006 25042456

[7] Singla M Kumar A Bal A Sarkar S Bhattacharyya S. Epithelial to mesenchymal transition induces stem cell like phenotype in renal cell carcinoma cells. Cancer Cell Int. (2018) 18:57. 10.1186/s12935-018-0555-6 29681769 PMC5896088

[8] Terry S Savagner P Ortiz-Cuaran S Mahjoubi L Saintigny P Thiery JP. insights into the role of EMT in tumor immune escape. Mol Oncol. (2017) 11:824–46. 10.1002/1878-0261.12093 28614624 PMC5496499

[9] Moll R Mitze M Frixen UH Birchmeier W. Differential loss of E-cadherin expression in infiltrating ductal and lobular breast carcinomas. Am J Pathol. (1993) 143:1731–42. 8256859 PMC1887260

[10] Oka H Shiozaki H Kobayashi K Inoue M Tahara H Kobayashi T., Expression of E-cadherin cell adhesion molecules in human breast cancer tissues and its relationship to metastasis. Cancer Res. (1993) 53:1696–701. 8453644

[11] Wei Z Shan Z Shaikh ZA. Epithelial-mesenchymal transition in breast epithelial cells treated with cadmium and the role of Snail. Toxicol Appl Pharmacol. (2018) 344:46–55. 10.1016/j.taap.2018.02.022 29501589 PMC5866788

[12] Polyak K Weinberg RA. Transitions between epithelial and mesenchymal states: acquisition of malignant and stem cell traits. Nat Rev Cancer. (2009) 9:265–73. 10.1038/nrc2620 19262571

[13] Kim HY Jackson TR Davidson LA. On the role of mechanics in driving mesenchymal-to-epithelial transitions. Semin Cell Dev Biol. (2017) 67:113–22. 10.1016/j.semcdb.2016.05.011 27208723 PMC5115991

[14] Katoch A Suklabaidya S Chakraborty S Nayak D Rasool RU Sharma D. Dual role of Par-4 in abrogation of EMT and switching on Mesenchymal to Epithelial Transition (MET) in metastatic pancreatic cancer cells. Mol Carcinog. (2018) 57:1102–15. 10.1002/mc.22828 29672923

[15] Pattabiraman DR Bierie B Kober KI Thiru P Krall JA Zill C. Activation of PKA leads to mesenchymal-to-epithelial transition and loss of tumor-initiating ability. Science. (2016) 351:aad3680. 10.1126/science.aad3680 26941323 PMC5131720

[16] Takaishi M Tarutani M Takeda J Sano S. Mesenchymal to epithelial transition induced by reprogramming factors attenuates the malignancy of cancer cells. PLoS ONE. (2016) 11:e0156904. 10.1371/journal.pone.0156904 27258152 PMC4892607

[17] Kalluri R Weinberg RA. The basics of epithelial-mesenchymal transition. J Clin Invest. (2009) 119:1420–8. 10.1172/JCI39104 19487818 PMC2689101

[18] Lee JM Dedhar S Kalluri R Thompson EW. The epithelial-mesenchymal transition: new insights in signaling, development, and disease. J Cell Biol. (2006) 172:973–81. 10.1083/jcb.200601018 16567498 PMC2063755

[19] Pasquier J Abu-Kaoud N Al Thani H Rafii A. Epithelial to mesenchymal transition in a clinical perspective. J Oncol. (2015). 2015:792182. 10.1155/2015/792182 26425122 PMC4575734

[20] Ye X Brabletz T Kang Y Longmore GD Nieto MA Stanger BZ. Upholding a role for EMT in breast cancer metastasis. Nature. (2017) 547:E1–3. 10.1038/nature22816 28682326 PMC6283276

[21] Marcucci F Stassi G De Maria R. Epithelial-mesenchymal transition: a new target in anticancer drug discovery. Nat Rev Drug Discov. (2016) 15:311–25. 10.1038/nrd.2015.13 26822829

[22] Mani SA Guo W Liao MJ Eaton EN Ayyanan A Zhou AY. The epithelial-mesenchymal transition generates cells with properties of stem cells. Cell. (2008) 133:704–15. 10.1016/j.cell.2008.03.027 18485877 PMC2728032

[23] Morel AP Lievre M Thomas C Hinkal G Ansieau S Puisieux A. Generation of breast cancer stem cells through epithelial-mesenchymal transition. PLoS ONE. (2008) 3:e2888. 10.1371/journal.pone.0002888 18682804 PMC2492808

[24] Wang J Wei Q Wang X Tang S Liu H Zhang FK. Transition to resistance: an unexpected role of the EMT in cancer chemoresistance. Genes Dis. (2016) 3:3–6. 10.1016/j.gendis.2016.01.002 28491932 PMC5421998

[25] Chakrabarty S Monlish DA Gupta M Wright TD Hoang VT Fedak M. Structure activity relationships of anthranilic acid-based compounds on cellular and *in vivo* mitogen activated protein kinase-5 signaling pathways. Bioorg Med Chem Lett. (2018) 28:2294–301. 10.1016/j.bmcl.2018.05.029 29803729

[26] Wu P Clausen MH Nielsen TE. Allosteric small-molecule kinase inhibitors. Pharmacol Ther. (2015) 156:59–68. 10.1016/j.pharmthera.2015.10.002 26478442

[27] Wang R Lv Q Meng W Tan Q Zhang S Mo X. Comparison of mammosphere formation from breast cancer cell lines and primary breast tumors. J Thorac Dis. (2014) 6:829–37. 10.3978/j.issn.2072-1439.2014.03.38 24977009 PMC4073404

[28] Cha B-K Kim Y-S Hwang K-E Cho K-H Oh S-H Kim B-R. Celecoxib and sulindac inhibit TGF-β1-induced epithelial-mesenchymal transition and suppress lung cancer migration and invasion via downregulation of sirtuin 1. Oncotarget. (2016) 7:57213. 10.18632/oncotarget.11127 27528025 PMC5302984

[29] Dong X Li R Xiu P Dong X Xu Z Zhai B. Meloxicam executes its antitumor effects against hepatocellular carcinoma in COX-2- dependent and -independent pathways. PLoS ONE. (2014) 9:e92864. 10.1371/journal.pone.0092864 24675684 PMC3968044

[30] Koo V El Mekabaty A Hamilton P Maxwell P Sharaf O Diamond J. Novel *in vitro* assays for the characterization of EMT in tumourigenesis. Cell Oncol. (2010) 32:67–76. 10.3233/CLO-2009-0501 20208135 PMC4619245

[31] Cao Z Livas T Kyprianou N. Anoikis and EMT: Lethal “Liaisons” during cancer progression. Crit Rev Oncogen. (2016) 21:155–68. 10.1615/CritRevOncog.2016016955 27915969 PMC5451151

[32] Dalvit C Invernizzi C Vulpetti A. Fluorine as a hydrogen-bond acceptor: experimental evidence and computational calculations. Chemistry Eur J. (2014) 20:11058–68. 10.1002/chem.201402858 25044441

[33] Gillis EP Eastman KJ Hill MD Donnelly DJ Meanwell NA. Applications of fluorine in medicinal chemistry. J Med Chem. (2015) 58:8315–59. 10.1021/acs.jmedchem.5b00258 26200936

[34] Meanwell NA. Fluorine and fluorinated motifs in the design and application of bioisosteres for drug design. J Med Chem. (2018) 61:5822–80. 10.1021/acs.jmedchem.7b01788 29400967

[35] Barros JC Marshall CJ. Activation of either ERK1/2 or ERK5 MAP kinase pathways can lead to disruption of the actin cytoskeleton. J Cell Sci. (2005) 118:1663–71. 10.1242/jcs.02308 15797923

[36] Fabbro D. 25 years of small molecular weight kinase inhibitors: potentials and limitations. Mol Pharmacol. (2015) 87:766–75. 10.1124/mol.114.095489 25549667

[37] Dar AC. A pickup in pseudokinase activity. Biochem Soc Trans. (2013) 41:987–94. 10.1042/BST20130110 23863168

[38] Eyers PA Murphy JM. Dawn of the dead: protein pseudokinases signal new adventures in cell biology. Biochem Soc Trans. (2013) 41:969–74. 10.1042/BST20130115 23863165

