# Supplementary figures and images for: Novel Diphenylamine Analogs Induce Mesenchymal to Epithelial Transition in Triple Negative Breast Cancer
Source: Front Oncol. 2019 Jul 30;9:672. doi: 10.3389/fonc.2019.00672 (PMC6682674; doi:10.3389/fonc.2019.00672)

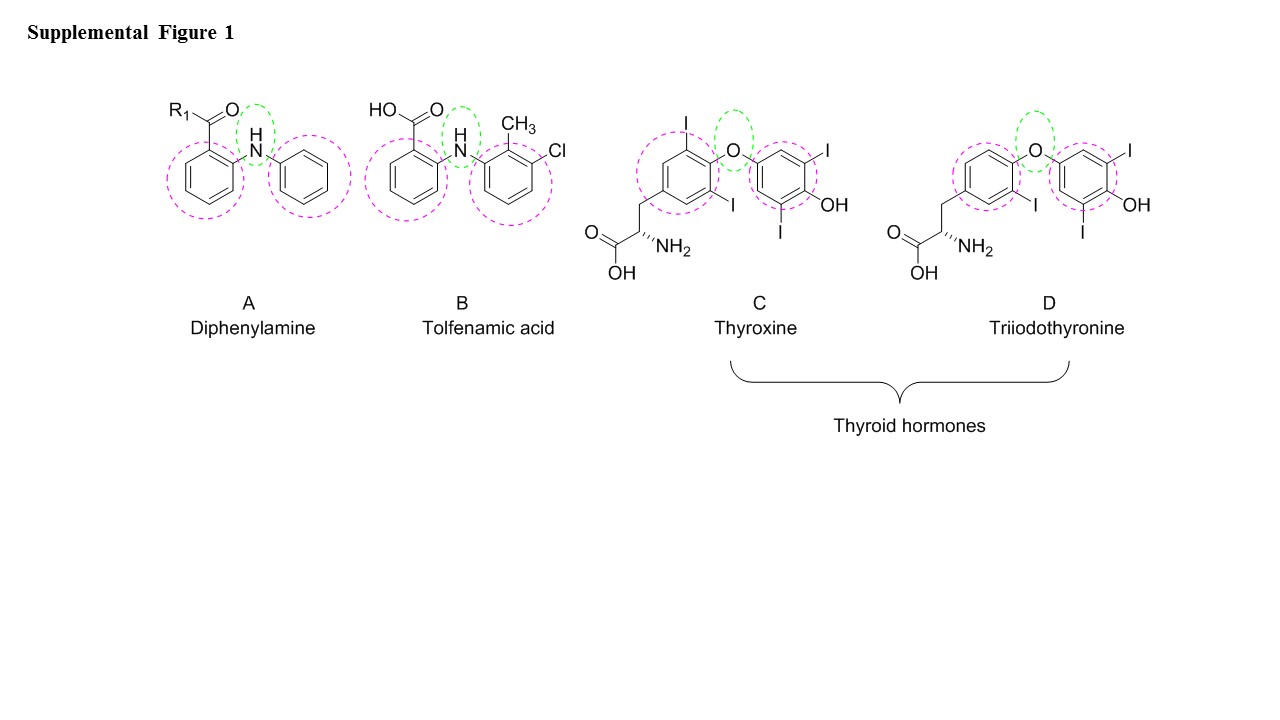

Supplement: Supplemental Figure 1 — Structural similarity between diphenylamines (A), tolfenamic acid (B), thyroxine (C), and triiodothyronine (D). [file Image_1.jpeg]

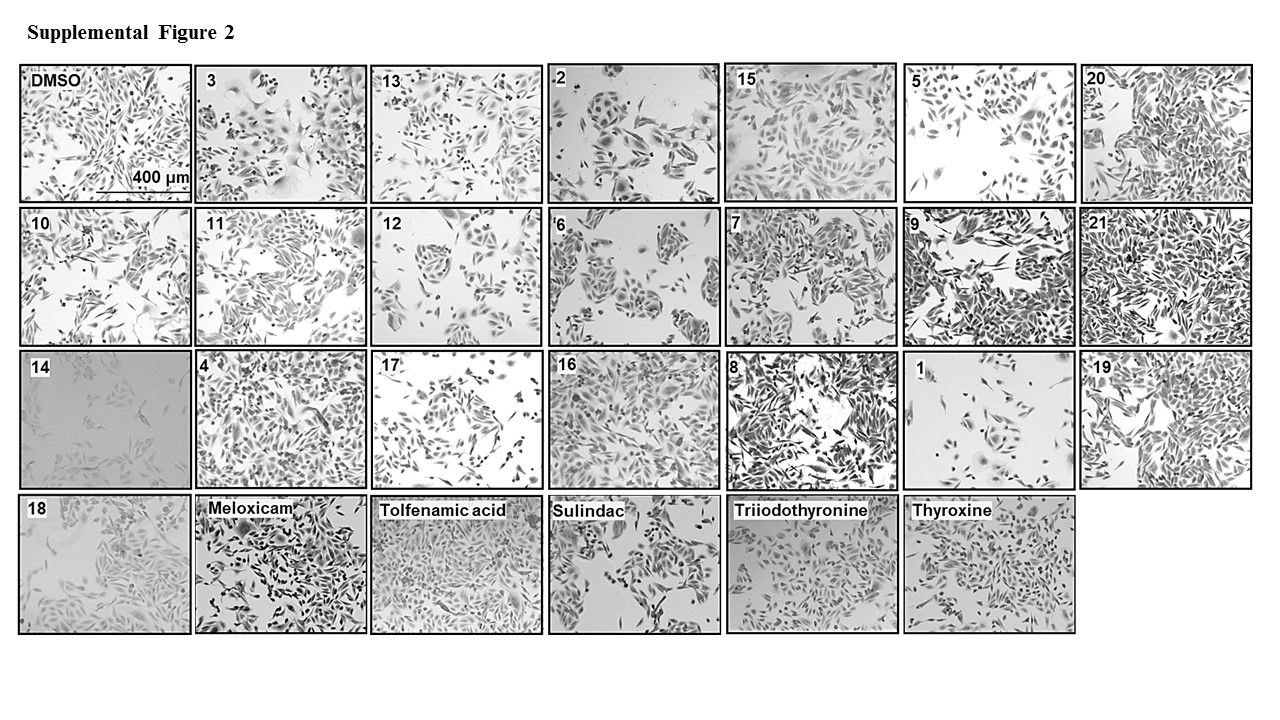

Supplement: Supplemental Figure 2 — Change in MDA-MB-231 cell morphology after treatment with diphenylamine derivatives for 5 days at 1 μM, as examined by crystal violet staining. [file Image_2.jpeg]

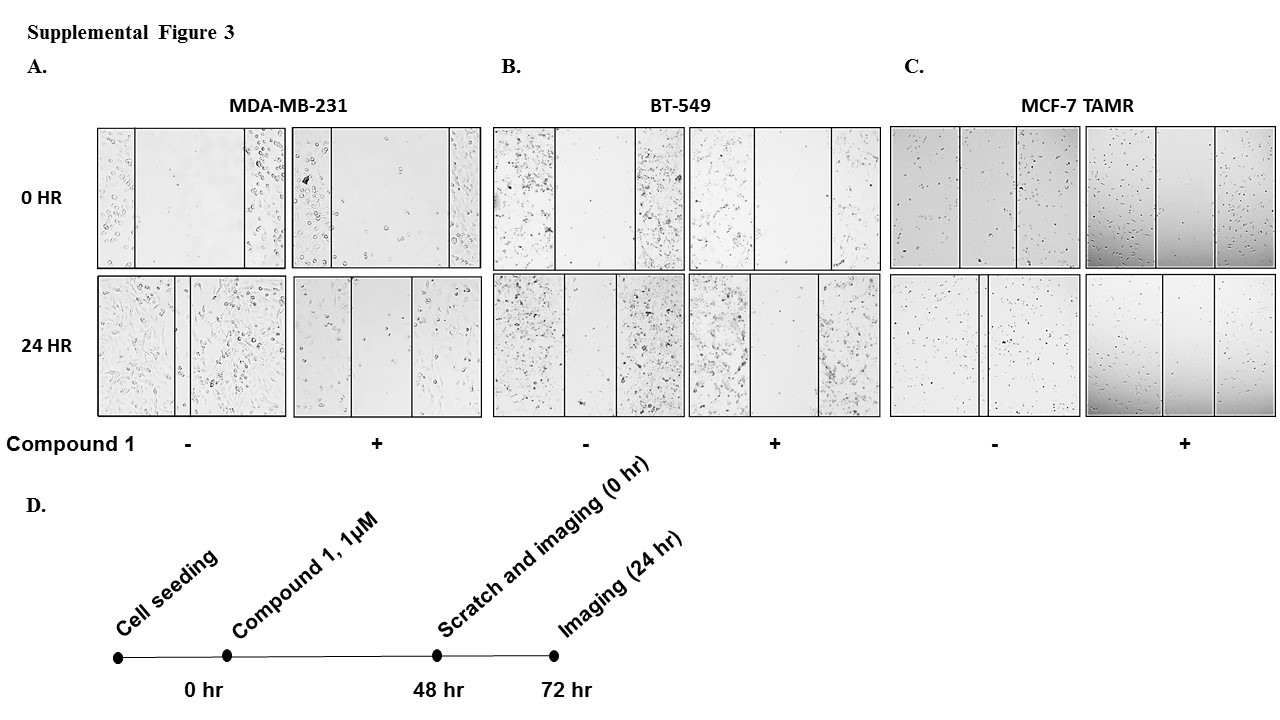

Supplement: Supplemental Figure 3 — Images of wound closure at the time of treatment and end-point. (A) MDA-MB-231 cells, (B) BT-549 cells (C) TAMR-MCF-7 cells (D) Scheme of treatment. [file Image_3.jpeg]

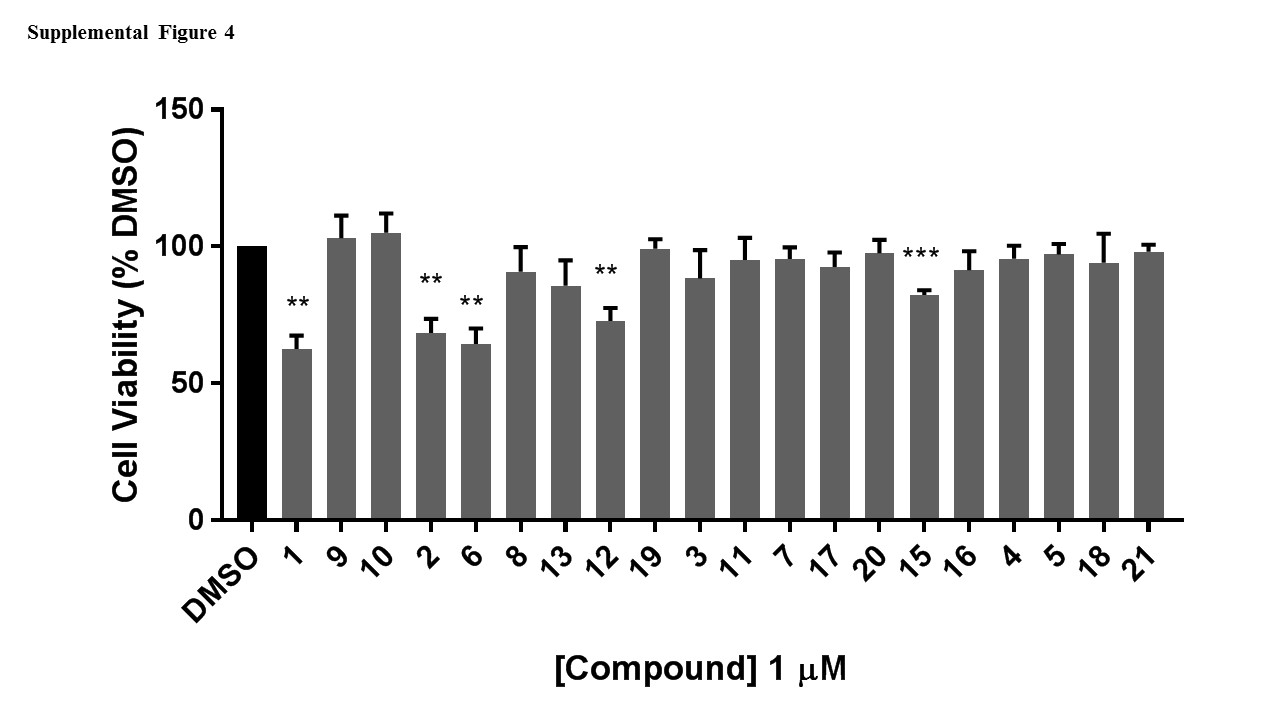

Supplement: Supplemental Figure 4 — Effect of diphenylamine analogs on cell viability in MDA-MB-231 cells. Data represent ± SEM of three different experiments. **p < 0.01; ***p < 0.001 vs. DMSO control group determined by unpaired two-tailed Student's t-test (n = 3). [file Image_4.jpeg]

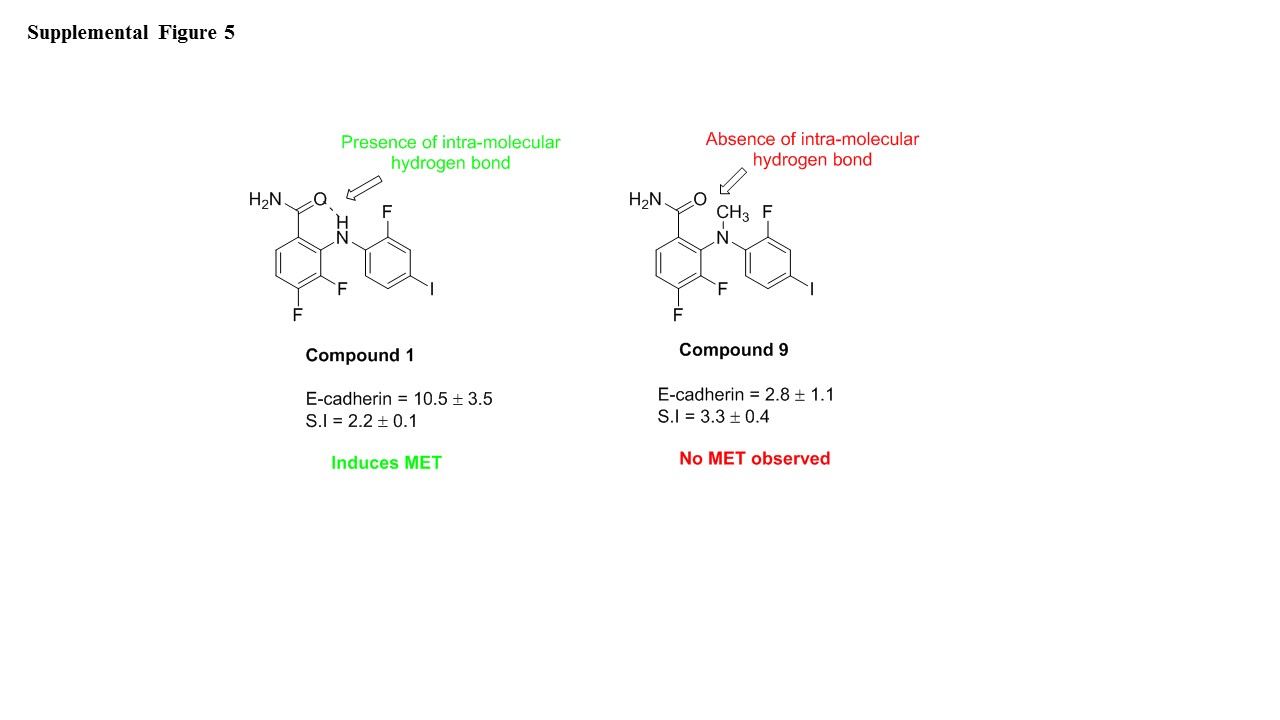

Supplement: Supplemental Figure 5 — Effect of intramolecular hydrogen bonding toward inducing MET. [file Image_5.jpeg]

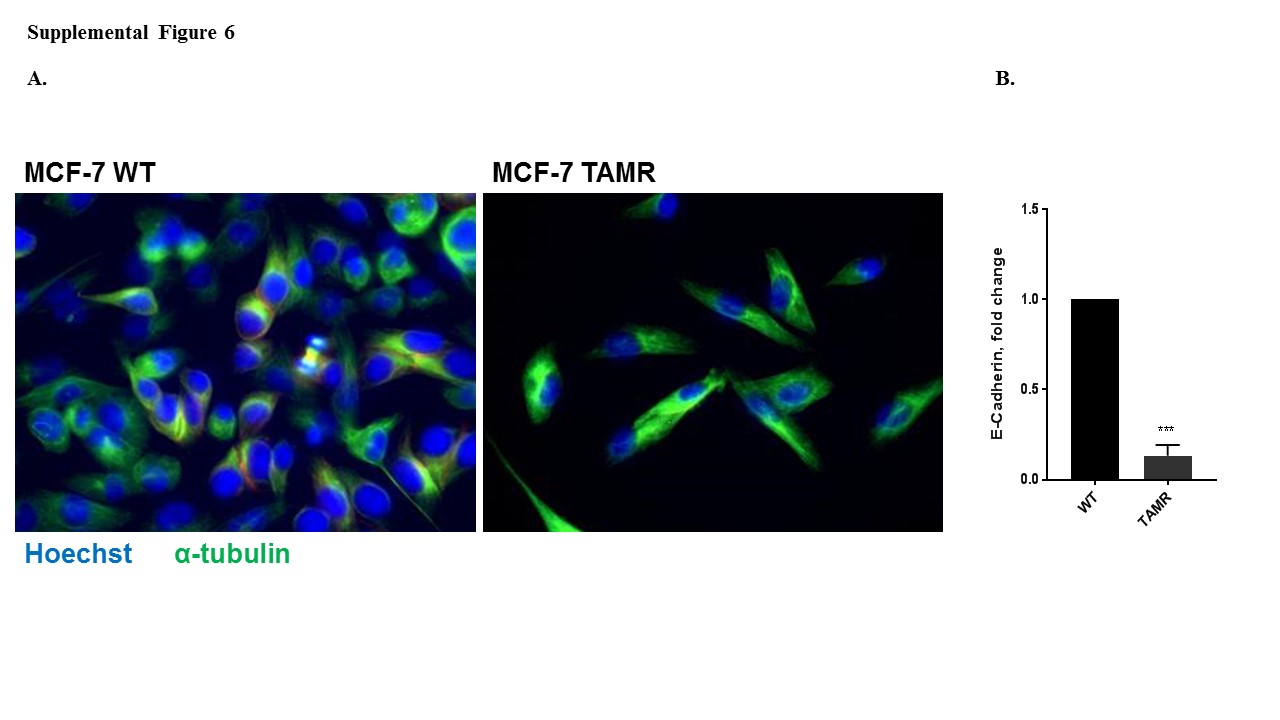

Supplement: Supplemental Figure 6 — (A) TAMR MCF-7 cells are mesenchymal in phenotype compared to the wild type MCF-7 cells. (B) E-cadherin protein expression is significantly decreased in TAMR MCF-7 cells compared to wildtype MCF-7 cells. ***p < 0.001 TAMR vs. wild type MCF-7 determined by two-tailed student's t-test. [file Image_6.jpeg]
