# Supplementary material for: Novel Diphenylamine Analogs Induce Mesenchymal to Epithelial Transition in Triple Negative Breast Cancer
Source: Front Oncol. 2019 Jul 30;9:672. doi: 10.3389/fonc.2019.00672 (PMC6682674; doi:10.3389/fonc.2019.00672)
Supplement: Supplemental Data Sheet 1 — Synthesis of diphenylamine analogs. [file Data_Sheet_1.PDF]

Synthesis of diphenylamine analogs

The synthesis of analogs **1-7**, **9-11**, **14-18** has been described in our previous communication [1]. The synthesis of analogs **8**, **12**, **13, and 19-21** is presented below.

2-((2-fluoro-4-iodophenyl)amino)benzamide (8)

A oven-dried round bottom flask was charged with 2-((2-fluoro-4-iodophenyl)amino)benzoic acid (**22**; 714 mg, 2 mmol) and 3 mL of dichloromethane. The reaction mixture was cooled with an ice-bath to 0 °C. 100 µL of anhydrous DMF was added followed by dropwise addition of oxalyl chloride (0.343 mL, 4 mmol) over 2 min. The ice-bath was removed after 15 minutes and the reaction was allowed to stir at 23 °C for 4 h. The solvent was removed and excess oxalyl chloride was azeotropically removed with 3 X 5 mL portions of dichloromethane under reduced pressure. The crude product (**23**) obtained, was dissolved in 7 mL of dichloromethane and treated with catalytic amount of DMAP (5 mg). The reaction mixture was cooled to 0 °C with ice-bath and 7N NH_3/MeOH (3 mL, 21 mmol) was added in a drop-wise manner. The ice bath was removed after 10 min and the reaction mixture was stirred at 23 °C for 24 h; completion of the reaction was determined by TLC. The solvent was removed, and the crude was dissolved in 20 mL of dichloromethane and washed with 5 mL of 1N NaOH, 2 X 25 mL water, 2 X 10 mL 0.5 N HCl, followed by brine wash, and dried over Na_2SO_4. The solvent was removed and the crude was purified with SiO_2 chromatography using 1:1 hexane/ethyl acetate to afford the final compound **8**, as a light brown powder 128 mg (18 %). MP = 244.1 - 246.2 ^{0}C. ^{1}H NMR (500 MHz, $CDCl_3$): δ 6.84-6.86 (d, 1 H, Ar), 7.21-7.25 (m, 1 H, Ar), 7.52-7.55 (m, 1 H, Ar), 7.63-7.66 (m, 1 H, Ar), 7.79-7.82 (m, 2 H, Ar), 8.20 (s, 1 H, NH), 8.40-8.42 (dd, 1 H, Ar). ESIMS (*m/z*): 355.0 $[M – H]^-$.

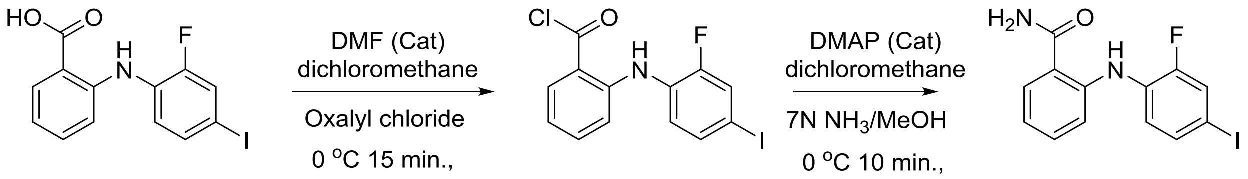

22 23 8

3,4-difluoro-2-((4-iodophenyl)amino)benzamide (12)

A oven-dried round bottom flask was charged with 2,3,4-trifluorobenzamide (**24**; 1.57 g, 9 mmol), 4-iodoaniline, (**25**; 2.16 g, 9.9 mmol), and 30 mL of anhydrous THF. The reaction mixture was cooled with an ice-bath to 0 °C and $LiNH_2$ (621 mg, 27 mmol) was added in 3 portions over a 10 min interval. The reaction was then warmed to an internal temperature of 62 °C and stirred for 12 h. The mixture was cooled to 0 °C and 1 N HCl was added maintaining the reaction internal temperature below 5 °C to yield a final pH of 1.0 (red to pHydrion paper). The reaction mixture was then extracted three times with 30 mL portions of ethyl acetate, washed three times with 5 mL 1 N HCl, brine, and then dried over Na_2SO_4. The extract was decanted and the solvent was removed under reduced pressure. The product was isolated on SiO_2 using 1:1 hexane/ethyl acetate to provide 1.85 g (55.2 %) of a pink-white solid **12**. MP = 188.6 °C. ^{1}H NMR (500 MHz, $CDCl_3$): δ 5.66-6.19 (bd, 2 H, NH_2), 6.67-6.71 (m, 2 H, Ar), 6.83-6.88 (m, 1 H, Ar), 7.39-7.42 (m, 1 H, Ar), 7.54-7.56 (dt, 2 H, Ar), 8.69 (s, 1 H, NH). ESIMS (*m/z*): 373.0 $[M – H]^-$.

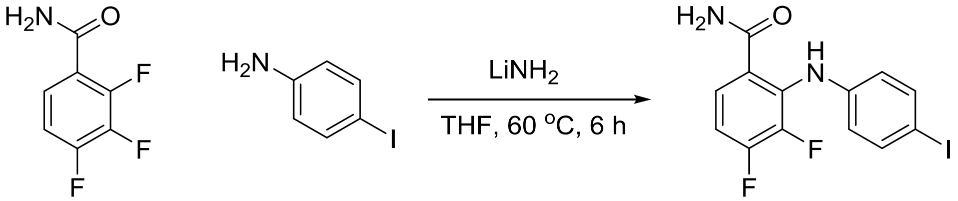

24 25 12

(3,4-difluoro-2-((2-fluoro-4-iodophenyl)amino)phenyl)(4-methylpiperazin-1-yl)methanone (13)

A oven-dried round bottom flask was charged with 3,4-difluoro-2-((2-fluoro-4-iodophenyl)amino)benzoic acid,[1] (**26**; 200 mg, 0.51 mmol) and 3 mL of dichloromethane. The reaction mixture was cooled on ice-bath to 0 °C. 100 µL of anhydrous DMF was added followed by dropwise addition of oxalyl chloride (0.086 mL, 1.02 mmol) over 5 min. The ice-bath was removed and the reaction was stirred at 23 °C for 4 h. The solvent was removed and excess oxalyl chloride was azeotropically removed with 3 X 5 mL portions of dichloromethane under reduced pressure. The crude product (**27**) was

dissolved into 3 mL of dichloromethane and *N*-methylpiperazine (0.3 mL, 2.55 mmol) was added at 0 °C. The reaction was stirred at 23 °C for 6 h; completion of the reaction was determined by TLC. A mixture of 10 mL of water and 10 mL of diethyl ether was added and the resultant mixture was extracted three times with 10 mL of diethyl ether, washed with 25 mL brine, and dried over Na_2SO_4. The extract was decanted, and then the solvent was removed under reduced pressure. The product was isolated on SiO_2 chromatography using 1:1 hexane/ethyl acetate followed by recrystallization from ethyl acetate furnishing **13** as a white solid 145.0 mg (60%). MP = 136.4 - 137.0 °C. ^{1}H NMR (400 MHz, $CDCl_3$): δ 1.41 (s, 1 H, NH). 1.57 (s, 3 H), 2.25 (m, 4 H), 3.39 (m, 2 H), 3.64 (m, 2 H), 6.56 (m, 1 H, Ar), 6.98 (m, 1 H, Ar), 7.05 (d, *J* = 1.6 Hz, 1 H, Ar), 7.29 (d, *J* = 1.6 Hz, 1 H, Ar), 7.41 (d, *J* = 1.6 Hz, 1 H, Ar). ESIMS (*m/z*): 476.1 [M + H]$^+$.

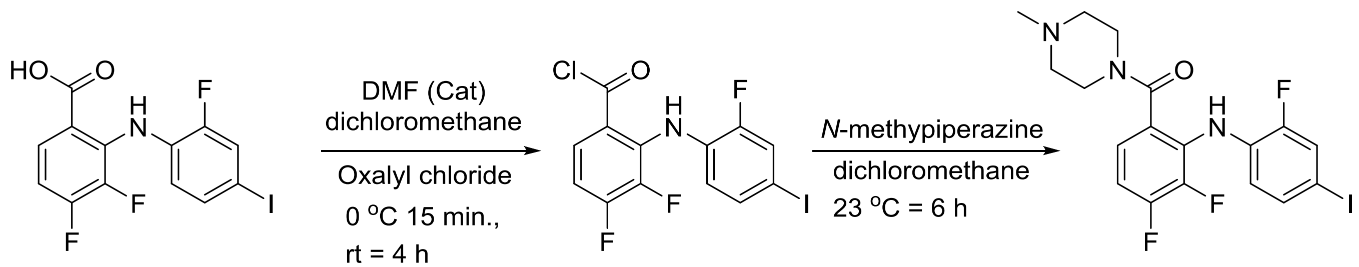

26 **27** **13**

(2-((3-azidophenyl)amino)-3,4-difluorophenyl)(4-methylpiperazin-1-yl)methanone (19)

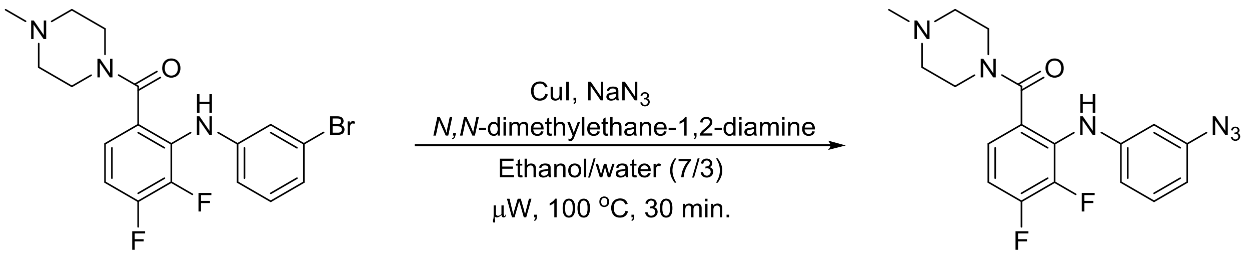

21 **19**

A dry 20 mL microwave reaction vessel was charged with **(**2-((3-bromophenyl)amino)-3,4-difluorophenyl)(4-methylpiperazin-1-yl)methanone, (**21**, 200.0 mg, 0.49 mmol), copper iodide, (9.5 mg, 0.05 mmol), sodium azide, (79.4 mg, 1.22 mmol), *N*,*N*-dimethylethane-1,2-diamine, (0.02 mL, 0.147 mmol), and 4 mL of a 7:3 mixture of ethanol/water. The microwave was programed to heat the reaction mixture to 100 °C for 30 minutes; progress of the reaction was monitored by TLC. The reaction mixture was extracted three times with 10 mL of dichloromethane. The solvent was removed under reduced pressure. The product was then isolated on SiO_2 (9:1 DCM/EtOH) to yield 114.3 mg (39%) of light black semi-solid. ^{1}H NMR (400 MHz, $CDCl_3$): δ 2.41 – 2.21 (m, 7H), 3.54 (bd, *J* = 97.6 Hz, 4H), 6.66 – 6.58 (m, 1H), 6.83 – 6.73 (m, 1H), 6.99 – 6.90 (m, 1H), 7.14 – 7.01 (m, 2H), 7.32 – 7.15 (m, 2H). ESIMS (*m/z*): 373.4 [M + H]$^+$.

2-((3-azidophenyl)amino)-N-ethyl-3,4-difluorobenzamide (**20**)

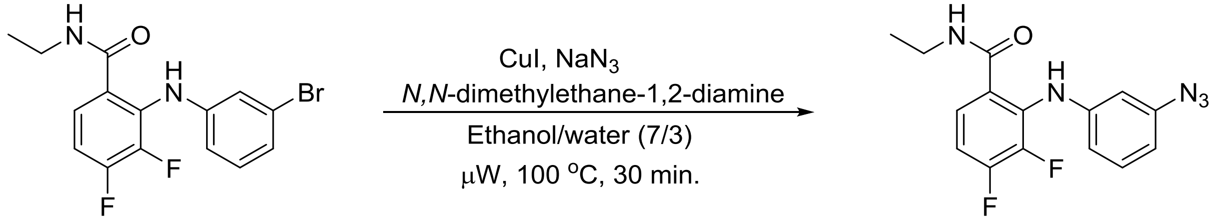

28 **20**

A dry 20 mL microwave reaction vessel was charged with 2-((3-bromophenyl)amino)-*N*-ethyl-3,4-difluorobenzamide, (**28**, 101.0 mg, 0.28 mmol), copper iodide, (6.6 mg, 0.03 mmol), sodium azide, (43.6 mg, 0.67 mmol), *N*,*N*-dimethyl ethyl amine, (9.0 µL, 0.084 mmol), and 1.2 mL of a 7:3 mixture of ethanol/water. The microwave was programed to heat the reaction mixture to 100 °C for 30 minutes. The reaction mixture was extracted three times with 10 mL of dichloromethane. The

solvent was removed under reduced pressure. The product was then isolated on SiO_2 (3:1 Hexanes/EA) to yield 15.3 mg (17%) of a white solid. ^{1}H NMR (400 MHz, $CDCl_3$): δ 1.21 (t, *J* = 7.3 Hz, 3H), 3.43 (qd, *J* = 7.2, 6.8, 5.1 Hz, 2H), 6.22 (s, 1H), 6.85 – 6.80 (m, 1H), 6.92 – 6.86 (m, 1H), 7.05 – 7.02 (m, 1H), 7.15 – 7.08 (m, 2H), 7.34 – 7.31 (m, 1H), 8.56 (s, 1H). ESIMS (*m/z*): 354.1 $[M + K – 2H]^-$.

(2-((3-bromophenyl)amino)-3,4-difluorophenyl)(4-methylpiperazin-1-yl)methanone (21)

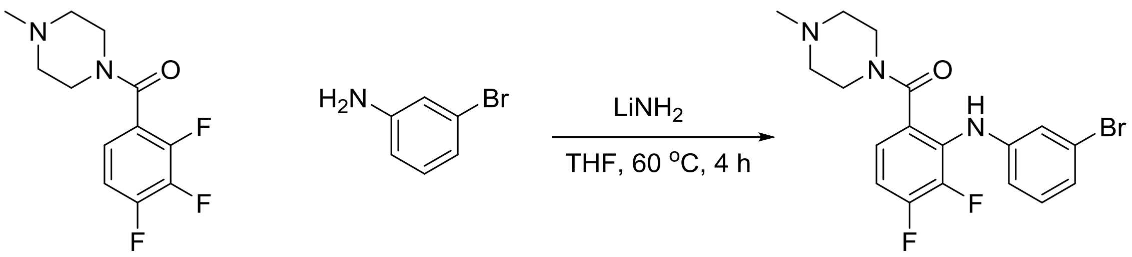

29 **21**

A dry 100 mL flask was cooled to 0 °C and charged with (4-methylpiperazin-1-yl)(2,3,4-trifluorophenyl)methanone, (**29**, 309.9 mg, 1.20 mmol), 3-bromoaniline, (0.13 mL, 1.19 mmol), and THF, (4 mL). Solid lithium amide, (88.4 mg, 3.85 mmol), was added in three portions over 10 minutes. The reaction was then warmed to 55 ° C (int. temp.) and stirred for 4 hours; progress of the reaction was monitored by TLC. The reaction mixture was extracted with 50 mL of Et_2O, washed with 10 mL of saturated brine two times, and then dried over Na_2SO_4. The solution was decanted and the solvent was removed under reduced pressure. The crude product was isolated on SiO_2 (89:9:1 DCM/EtOH/TEA) to give 425.2 mg (87 %) of light brown semi-solid. ^{1}H NMR (400 MHz, $CDCl_3$) δ 2.33 – 2.14 (m, 7H), 3.65 – 3.24 (m, 4H), 6.70 – 6.64 (m, 1H), 6.98 – 6.83 (m, 5H), 6.70 – 6.64 (m, 1H). ESIMS (*m/z*): 410.2 $[M + H]^+$.

[1] S. Chakrabarty, D.A. Monlish, M. Gupta, T.D. Wright, V.T. Hoang, M. Fedak, I. Chopra, P.T. Flaherty, J. Madura, S. Mannepelli, M.E. Burow, J.E. Cavanaugh, Structure activity relationships of anthranilic acid-based compounds on cellular and in vivo mitogen activated protein kinase-5 signaling pathways, Bioorg. & Med. Chem. Lett., 28 (2018) 2294-2301.
